# Supplementary material for: Targeting CX3CR1 Suppresses the Fanconi Anemia DNA Repair Pathway and Synergizes with Platinum
Source: Cancers (Basel). 2021 Mar 22;13(6):1442. doi: 10.3390/cancers13061442 (PMC8004634; doi:10.3390/cancers13061442)
Supplement: Supplementary file 1 [file cancers-13-01442-s001.pdf]

cancers

# Supplementary Materials: Targeting CX3CR1 suppresses the Fanconi Anemia DNA repair pathway and synergizes with platinum

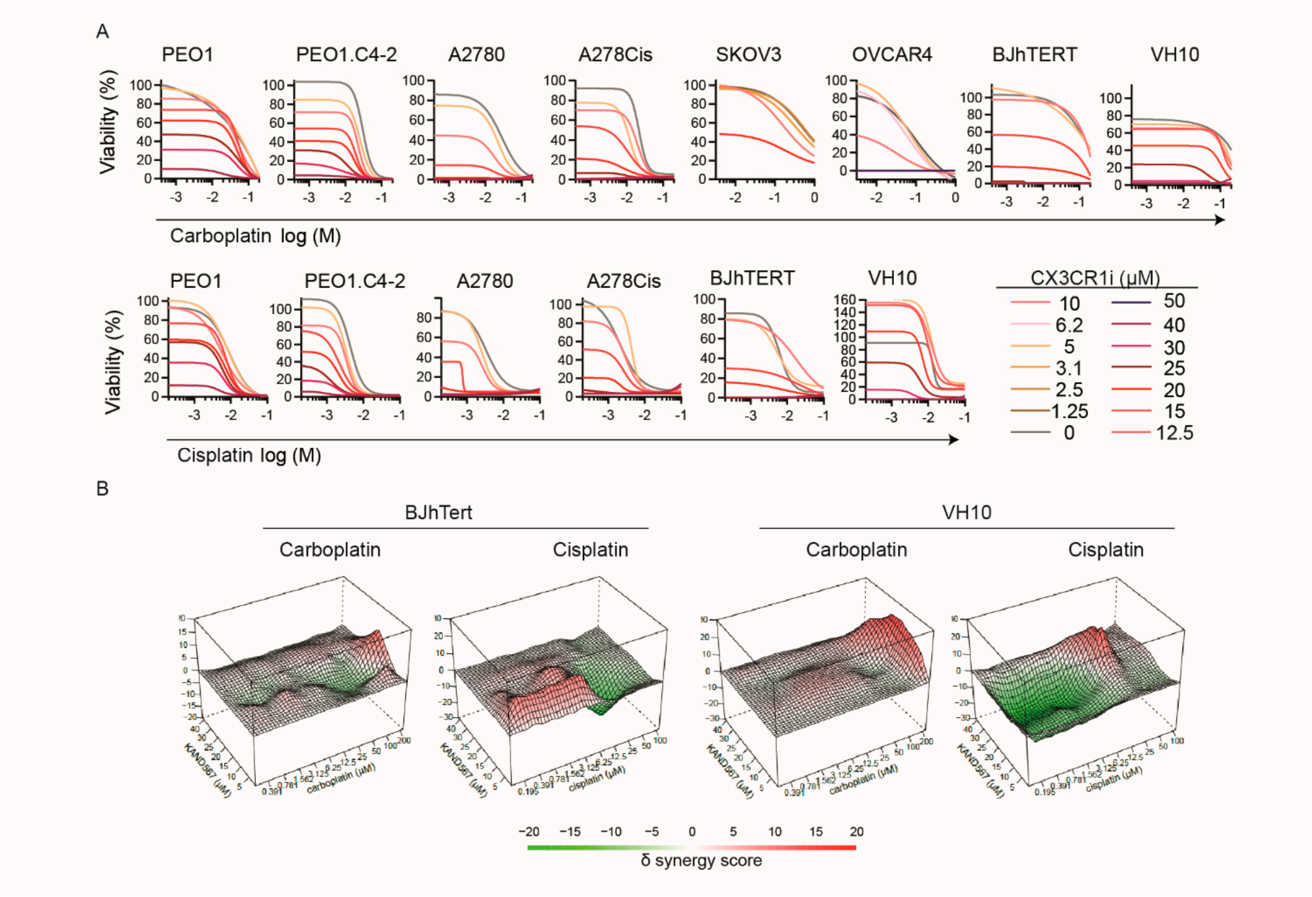


**Figure S1.** Synergy studies upon CX3CR1i and platinum combination treatments. (a) Representative dose-response curves from Figure 1a (b) representative 3D synergy maps upon CX3CR1i combination with carboplatin or cisplatin in non-transformed BJhTERT and VH10 cell lines from Figure 1b.

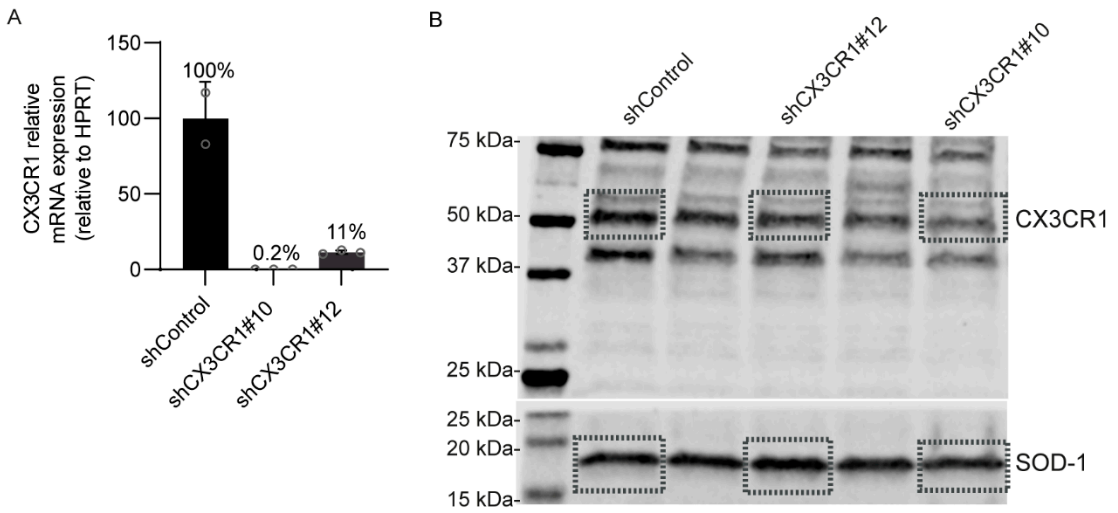


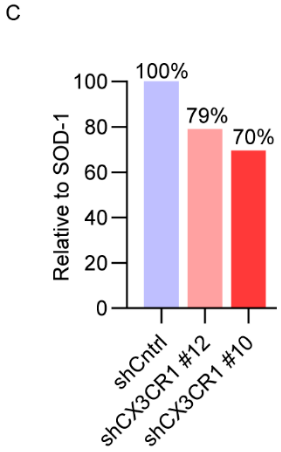


Densitometry Readings

| | CX3CR1/SOD-1 |
|---|---|
| shControl | 0.133251834 |
| shCX3CR1 #12 | 0.105228758 |
| shCX3CR1 #10 | 0.092622951 |

**Figure S2.** Validation of shCX3R1 in A2780 cells. (a) Relative CX3CR1 mRNA levels of A2780 shCX3CR1 and shControl cell lines normalised to shControl from Figure 1C and D. (b) Western blot showing CX3CR1 protein levels in A2780 shCX3CR1 and shControl cell lines. **c)** Quantification of CX3CR1 protein expression from (b) measured by Western blot densitometry, normalized to SOD-1.

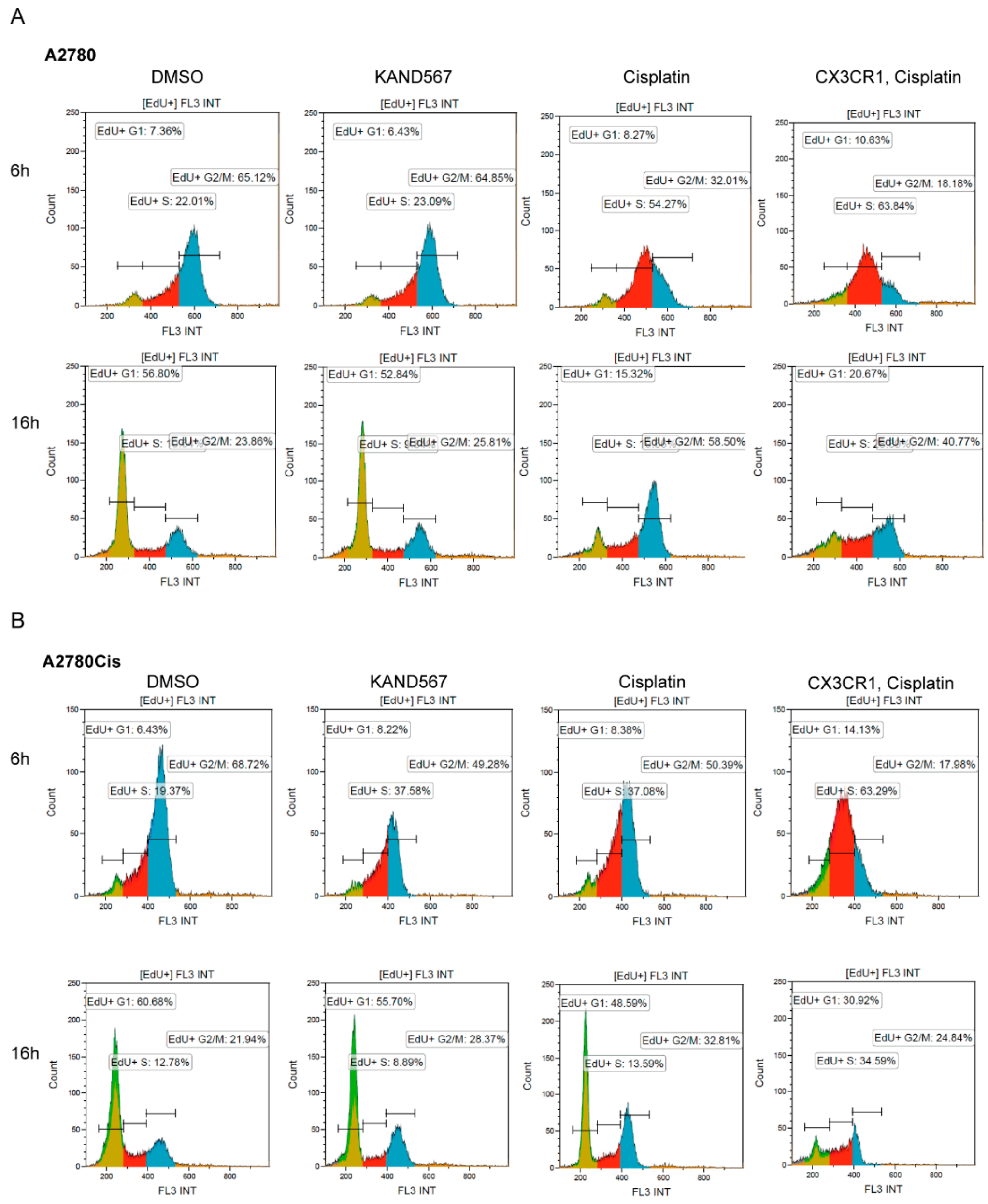


**Figure S3. (a, b)** Histograms representing cell cycle distribution (yellow: G1; red: S; blue: G2/M) of EdU positive A2780 (a) and A2780Cis (b) cells treated with cisplatin and CX3CR1i alone and in combination treatments in Figure 2A, B. X axis: propidium iodide intensity.

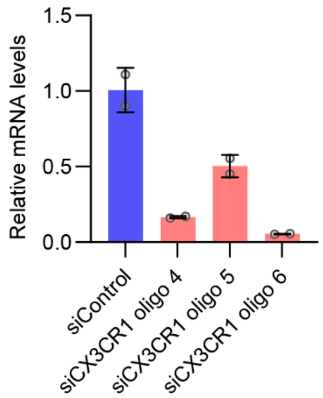


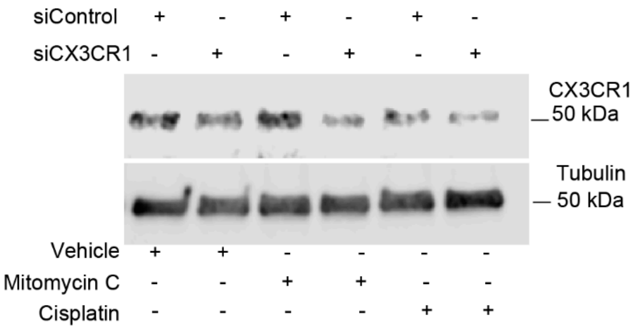


C

Densitometry Readings

| | **CX3CR1/Tubulin** |
|---|---|
| siControl, Vehicle | 0.01111628 |
| siCX3CR1, Vehicle | 0.00884393 |
| siControl, MMC | 0.01030303 |
| siCX3CR1, MMC | 0.00732620 |
| siControl, Vehicle | 0.00801453 |
| siCX3CR1, Vehicle | 0.00560158 |

**Figure S4.** Validation of siCX3CR1 knockdown. **(a)** Relative CX3CR1 mRNA levels normalised to non-targeting control upon 72 h of CX3CR1-targeting siRNA oligo #4,# 5 and #6 in U2OS cells. **(b)** Western blot showing CX3CR1 protein levels upon 72 h of CX3CR1-targeting oligo pool (#4-6) or siControl in U2OS cells treated as in Figure 3d. $\alpha$-Tubulin was used as loading control. **(c)** Quantification of CX3CR1 protein expression from (b) measured by Western blot densitometry, normalized to $\alpha$-tubulin.

A

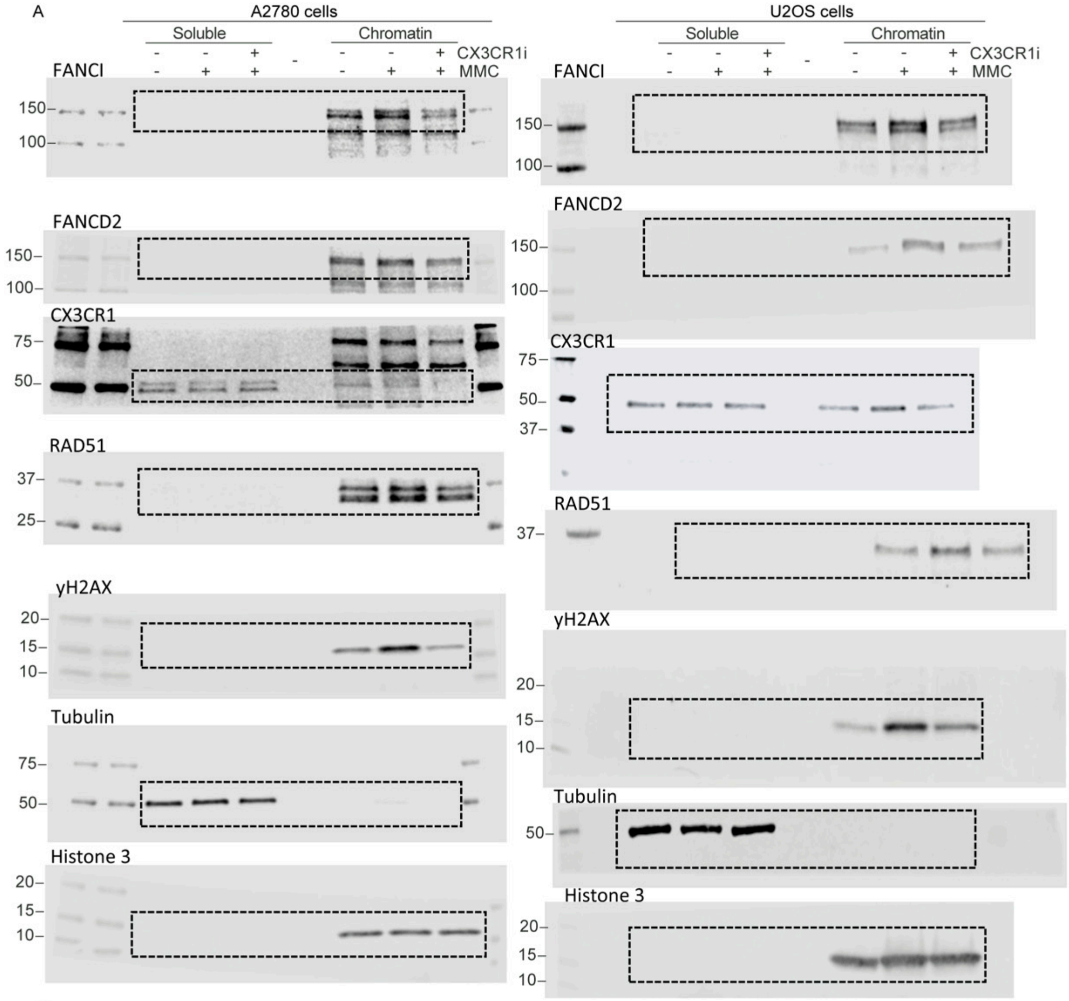


B

| | FANCI/H3 | FANCD2/H3 | RAD51/H3 | γH2AX/H3 | CX3CR1/H3 | CX3CR1/Tubulin (soluble) |
|---|---|---|---|---|---|---|
| A2780, Vehicle | 0.01691689 | 0.03887399 | 0.16729223 | 0.14959786 | 0.02037534 | 0.00178636 |
| A2780, Mitomycin C | 0.02053333 | 0.04506667 | 0.20373333 | 0.29866667 | 0.03200000 | 0.00128571 |
| A2780, Mitomycin C + CX3CR1i | 0.01056000 | 0.02933333 | 0.13360000 | 0.09893333 | 0.02602667 | 0.00162189 |
| U2OS, Vehicle | 0.03759434 | 0.00924528 | 0.00575472 | 0.01915094 | 0.108018868 | 1.919463087 |
| U2OS, Mitomycin C | 0.04047619 | 0.01920635 | 0.00769841 | 0.07420635 | 0.131746032 | 2.75 |
| U2OS, Mitomycin C + CX3CR1i | 0.02989286 | 0.01278571 | 0.00471429 | 0.03571429 | 0.066785714 | 2.1 |

C

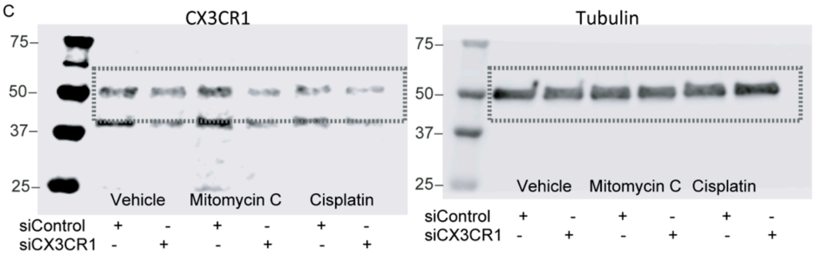


**Figure S5.** CX3CR1 modulates recruitment of key FA repair factors upon ICL induction. **(a)** Uncropped western blot membranes, dotted lines indicate the cut sections used in Figure 4D western blot. **(b)** Quantification of protein expression from (a) measured by Western blot densitometry, normalized to histone 3 (H3) or Tubulin. **(c)** Uncropped western blot membranes, dotted lines indicate the cut sections used in Figure S4b western blot.

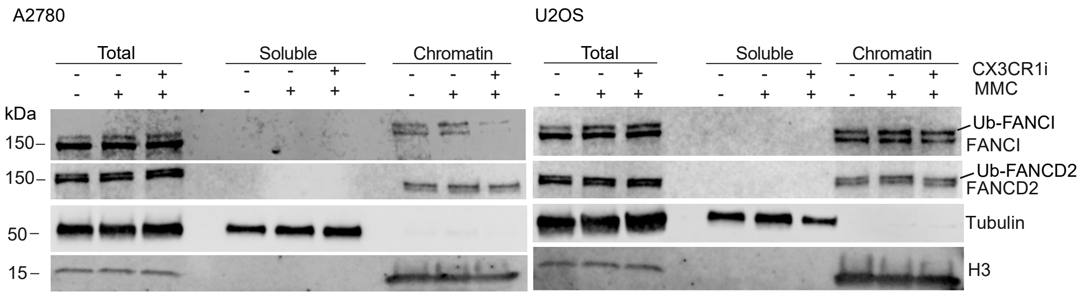


**Figure S6. CX3CR1 modulates recruitment of FANCI and FANCD2 upon ICL induction.** A2780 (left panel) or U2OS (right panel) cells were synchronized at the G1/S boundary and released for 3 h into vehicle, 120 ng/mL mitomycin C with or without 10 µM CX3CR1i followed by immunoblot of the soluble and nuclease-insoluble chromatin fractions, *n*=2. Images of the uncropped Western blots can be found in Figures S7.

A

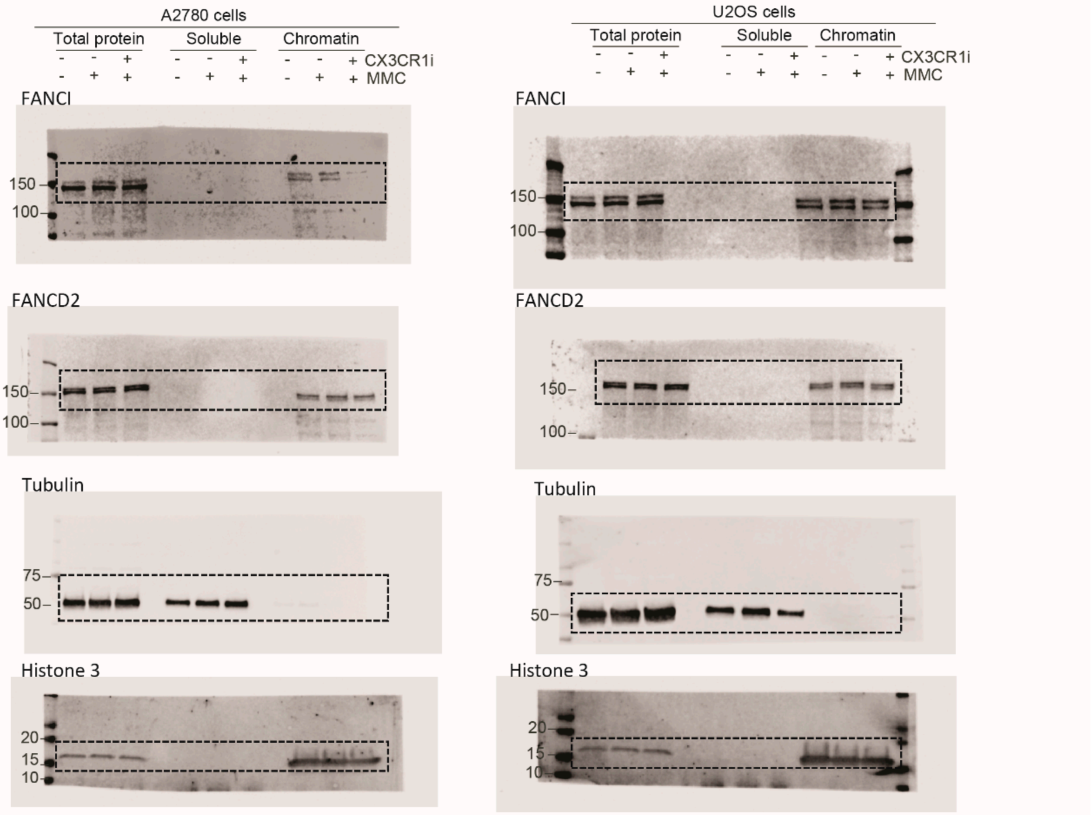


B

Densitometry readings of chromatin-bound proteins over Histone 3

| | FANCI | Ub-FANCI | FANCD2 |
|---|---|---|---|
| A2780, Vehicle | 0.005247 | 0.005989 | 0.02467 |
| A2780, Mitomycin C | 0.005933 | 0.01194 | 0.037313 |
| A2780, Mitomycin C + CX3CR1i | 0.002452 | 0.005274 | 0.032842 |
| U2OS, Vehicle | 0.020548 | 0.016781 | 0.036986 |
| U2OS, Mitomycin C | 0.026867 | 0.029905 | 0.057278 |
| U2OS, Mitomycin C + CX3CR1i | 0.015609 | 0.019057 | 0.041609 |

Densitometry readings of total proteins over Tubulin

| | FANCI | Ub-FANCI | FANCD2 |
|---|---|---|---|
| A2780, Vehicle | 0.012952 | 0.002171 | 0.02 |
| A2780, Mitomycin C | 0.011359 | 0.002524 | 0.01932 |
| A2780, Mitomycin C + CX3CR1i | 0.008 | 0.003304 | 0.016741 |
| U2OS, Vehicle | 0.014222 | 0.004067 | 0.023926 |
| U2OS, Mitomycin C | 0.012803 | 0.006091 | 0.021742 |
| U2OS, Mitomycin C + CX3CR1i | 0.010909 | 0.005662 | 0.020195 |

**Figure S7. CX3CR1 modulates recruitment of FANCI and FANCD2 upon ICL induction (a)** Uncropped western blot membranes, dotted lines indicate the cut sections used in Figure S6 western blot. **(b)** Quantification of protein expression from (a) measured by Western blot densitometry, normalized to histone 3 (H3) or Tubulin.

**Table S1.** Antibodies used in the study.

| Antibody | Source | Catalog # | Dilution |
|---|---|---|---|
| p-H2A.X S139 | Cell Signaling | 2577s | 1:500, WB |
| p-H2A.X S139 | Millipore | 05-636 | 1:500, WB |
| α-tubulin | Abcam | ab7291 | 1:10,000, WB |
| SOD-1 | Santa Cruz | sc-17767 | 1:10,000, WB |
| CX3CR1 | Abcam | ab8021 | 1:500, WB; 1:250 IF |
| Histone 3 | Abcam | ab18521 | 1:5000, WB |
| FANCD2 | Santa Cruz | sc-20022 | 1:400, WB; 1:250 IF |
| FANCI | Santa Cruz | sc-271316 | 1:400, WB |
| RAD51 | Santa Cruz | sc-8349 | 1:400, WB |
| Anti-Cisplatin modified antibody | Abcam | ab103261 | 1:200, flow cytometry |
| BrdU (anti-rat) | BioRad | MCA6144 | 1:1000, DNA fiber assay |
| IdU (anti-mouse) | Biosciences | 347580 | 1:1000, DNA fiber assay |
| IRDye 680RD Donkey anti-Mouse IgG | Li-Cor Biosciences | 925-68072 | 1:10000, WB |
| IRDye 800CW Donkey anti-Rabbit IgG | Li-Cor Biosciences | 925-32213 | 1:10000, WB |
| RDye 680CW Donkey anti-Rabbit IgG | Li-Cor Biosciences | 925-68073 | 1:10000, WB |
| IRDye 800CW Donkey anti-Mouse IgG | Li-Cor Biosciences | 926-32212 | 1:10000, WB |
| Donkey anti-mouse Alexa Fluor 488 | Invitrogen | A-21202 | 1:500, IF |
| Donkey anti-rabbit Alexa Fluor 568 | Invitrogen | A-10042 | 1:500, IF |
| Donkey anti-mouse Alexa Fluor 488 | Invitrogen | A-21202 | 1:500, DNA fiber assay |
| Goat anti-rat Alexa Fluor 568 | Invitrogen | A-11077 | 1:500, DNA fiber assay |
| Goat anti-rat Alexa Fluor 647 | Invitrogen | A-21247 | 1:50, flow cytometry |

**Table S2.** Sequences of siRNAs targeting CX3CR1 used in the study.

| CX3CR1 siRNAs (Qiagen 1027416) | Catalog no. | Lot no. | Target sequence |
|---|---|---|---|
| #4 | SI00025564 | 201712220191 | AACTCAGACTACTTTAGTTAA |
| #5 | SI02626008 | 201712220191 | TCAGATGTGGTAACTGTTAAA |
| #6 | SI03064383 | 201612080692 | CAGAGTGATAGCCTTCCGTAA |

**Table S3.** CX3CR1 shRNA hairpins from Sigma Mission library (TRCMm1.0).

| ID | Full sequence | Sense sequence |
|---|---|---|
| TRCN0000011310 (shCX3CR1#10) | CCGGGCCTTGTCTGATCTGCTGTTTCTCGA-GAAACAGCAGATCAGACAAGGCTTTTT | GCCTTGTCTGATCTGCTGTTT |
| TRCN0000011312 (shCX3CR1#12) | CCGGCGGTTGCATTTAGCCATTGTTCTCGA-GAACAATGGCTAAATGCAACCGTTTTT | CGGTTGCATTTAGCCATTGTT |
